# Different Activation Mechanisms of Excitatory Networks in the Rat Oculomotor Integrators for Vertical and Horizontal Gaze Holding

**DOI:** 10.1523/ENEURO.0364-19.2019

**Published:** 2020-01-02

**Authors:** Yasuhiko Saito, Taketoshi Sugimura

**Affiliations:** Department of Neurophysiology, Nara Medical University, Kashihara 634-8521, Japan

**Keywords:** excitatory network, gaze holding, integrator, rat, slice patch clamp, synaptic mechanism

## Abstract

Gaze holding in the horizontal and vertical directions is separately controlled via the oculomotor neural integrators, the prepositus hypoglossi nucleus (PHN) and the interstitial nucleus of Cajal (INC), respectively. Our previous *in vitro* studies demonstrated that transient, high-frequency local stimulation of the PHN and the INC increased the frequency of spontaneous EPSCs that lasted for several seconds. The sustained EPSC response of PHN neurons was attributed to the activation of local excitatory networks primarily mediated via Ca^2+^-permeable AMPA (CP-AMPA) receptors and Ca^2+^-activated nonselective cation (CAN) channels. However, the contribution of CP-AMPA receptors to the activation of INC excitatory networks appeared to be small. In this study, we clarified the mechanisms of excitatory network activation in the PHN and INC using whole-cell recordings in rat brainstem slices. Although physiological and histological analyses showed that neurons that expressed CP-AMPA receptors existed not only in the PHN but also in the INC, the effect of a CP-AMPA receptor antagonist on the sustained EPSC response was significantly weaker in INC neurons than in PHN neurons. Meanwhile, the effect of an NMDA receptor antagonist on the sustained EPSC response was significantly stronger in INC neurons than in PHN neurons. Furthermore, the current and the charge transfer mediated via NMDA receptors were significantly larger in INC neurons than in PHN neurons. These results strongly suggest that these excitatory networks are activated via different synaptic mechanisms: a CP-AMPA receptor and CAN channel-dependent mechanism and an NMDA receptor-dependent mechanism in horizontal and vertical integrators, respectively.

## Visual Abstract

**Figure:**
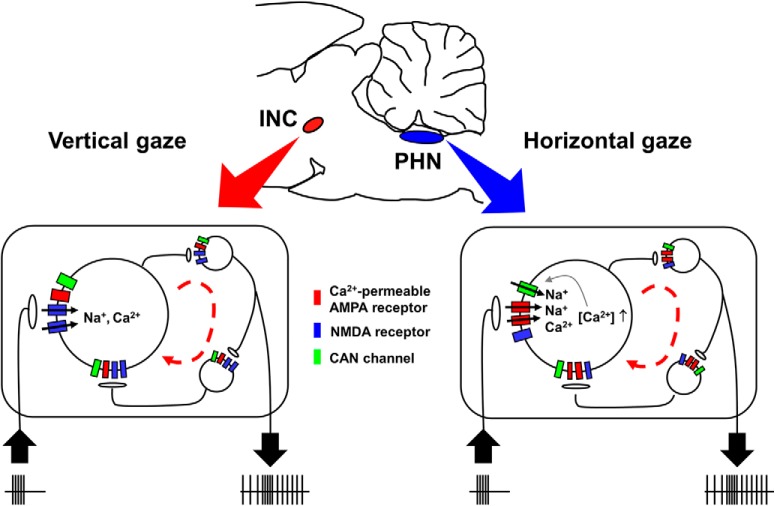

## Significance Statement

Position signals necessary for gaze holding are produced from velocity signals by mathematical integration via brainstem oculomotor integrators, which are separated into horizontal and vertical integrators. Although excitatory networks in the integrators are essential for their functions, the mechanisms of sustained activation in their networks have not been clarified. This study reports that the activation of excitatory networks in the horizontal integrator is mediated predominantly via Ca^2+^-permeable AMPA receptors and Ca^2+^-activated nonselective cation channels, while their activation in the vertical integrator is mediated predominantly via NMDA receptors. This result strongly suggests that excitatory networks are activated via different synaptic mechanisms between horizontal and vertical integrators.

## Introduction

The visual system does not have a high temporal resolution, and, therefore, holding the eye on a visual target for a certain amount of time is necessary for effective operation of the system. The function of gaze holding is executed primarily by an oculomotor neural integrator that converts transient burst signals that are proportional to eye or head velocity into sustained signals that are proportional to eye position (Skavenski and Robinson, 1973; Robinson, 1975, 1989; Fukushima, 1987, 1991; Fukushima et al., 1992; Fukushima and Kaneko, 1995; Moschovakis, 1997; Leigh and Zee, 2015). Although the generation of sustained signals necessary for gaze holding is attributed to neural network mechanisms, such as positive feedback excitation through mutual inhibition and/or recurrent excitation (Cannon et al., 1983; Galiana and Outerbridge, 1984; Cannon and Robinson, 1985; Draye et al., 1997; Arnold and Robinson, 1997; Seung et al., 2000), there are few experimental studies on the synaptic mechanisms employed to produce sustained signals in the mammalian neural integrator (Navarro-López et al., 2004, 2005; Joshua and Lisberger 2015).

Horizontal gaze holding and vertical gaze holding are separately controlled by different oculomotor neural integrators, the prepositus hypoglossi nucleus (PHN) and the interstitial nucleus of Cajal (INC), respectively (Fukushima, 1987, 1991; Fukushima et al., 1992; Fukushima and Kaneko, 1995; Moschovakis, 1997; Leigh and Zee, 2015). We previously showed that under a blockade of inhibitory synaptic transmission, the application of a transient (0.2 s), high-frequency (100 Hz) electrical stimulation to a nearby site of a recorded PHN neuron induced an increase in the frequency of spontaneous EPSCs that lasted for several seconds (Saito and Yanagawa, 2010). This result indicates the presence of local excitatory networks in the PHN, which maintain sustained activity responsible for the generation of eye position signals. Pharmacological analyses have indicated that the sustained EPSC response is attributed preferentially to the activation of Ca^2+^-permeable AMPA (CP-AMPA) receptors and Ca^2+^-activated nonselective cation (CAN) channels of PHN neurons.

In contrast to the accumulating findings regarding the PHN, there has been no report regarding the local networks in the INC. In our recent study (Saito et al., 2017), we examined excitatory networks in the INC by using the same experimental procedure used in the PHN study and found that the sustained EPSC responses also occurred in the INC. However, the effect of a CP-AMPA receptor antagonist on the sustained EPSC response was significantly smaller in the INC than in the PHN. These results suggest that the excitatory networks responsible for the sustained EPSC response are also present in the INC; however, the activation mechanism of the INC networks differs from the CP-AMPA receptor-dependent mechanism used in the PHN. In this study, we clarified the synaptic mechanisms preferential to the activation of excitatory networks in the PHN and the INC using whole-cell recordings in rat brainstem slices.

## Materials and Methods

All experimental procedures were approved by the Animal Care Committee of Nara Medical University, and the experiments were conducted in accordance with the guidelines outlined by the US National Institutes of Health regarding the care and use of animals for experimental research. Although our previous studies were performed in slice preparations obtained from Wistar rats (Saito and Yanagawa, 2010; Saito et al., 2017), we used slices obtained from Long–Evans rats of either sex in this study because pigmented rats have higher visual acuity than albino rats (Birch and Jacobs, 1979; Prusky et al., 2002). A total of 30 Long–Evans rats (18–21 postnatal days old) were used in this study.

### Slice preparation and whole-cell recording

The slice preparation and whole-cell patch-clamp recording procedures were similar to those described previously (Saito and Yanagawa, 2010; Saito et al., 2017). Briefly, the rat was deeply anesthetized with isoflurane and decapitated. The adequacy of the depth of anesthesia was judged by the absence of reflex movements to toe pinches. Frontal brain slices that included the INC or the PHN were cut using a Microslicer (Pro 7, Dosaka EM) in ice-cold sucrose solution containing the following (in mm): 234 sucrose, 2.5 KCl, 1.25 NaH_２_PO_４_, 10 MgSO_４_, 0.5 CaCl_2_, 26 NaHCO_3_, and 11 glucose, bubbled with 95% O_2_ and 5% CO_2_. The thicknesses of the slices were 400 μm for the recording of sustained EPSC responses and 250 μm for recordings of current responses to the local application of agonists. The slices were recovered in an interface-type chamber perfused with an extracellular solution containing the following (in mm): 125 NaCl, 2.5 KCl, 2 CaCl_2_, 1 MgCl_2_, 1.25 NaH_2_PO_4_, 26 NaHCO_3,_ and 25 glucose, aerated with 95% O_2_ and 5% CO_2_, pH 7.4, at 33°C for 1 h. After recovery, the slices were incubated in an aerated extracellular solution at room temperature. For recordings, each slice was placed in a submerged recording chamber on an upright microscope (model DM LFS, Leica Microsystems; RRID:SCR_008960) and continuously perfused with the extracellular solution at a rate of 3 ml/min. The bath temperature was maintained at 30–32°C using an in-line heater (SH-27A, Warner Instruments). Whole-cell recordings were performed using an EPC-8 patch-clamp amplifier (HEKA), and the data were acquired using a pClamp9 system (Molecular Devices). The internal solution contained the following (in mm): 145 Cs-gluconate, 5 CsCl, 0.2 EGTA, 2 Mg-ATP, 0.3 Na-GTP, 10 HEPES, 0.1 spermine, and 5 lidocaine *N*-ethyl bromide (QX-314) at pH 7.3, and its osmolarity was 280–290 mOsm/L. The resistance of patch electrodes was 4–8 MΩ in the bath solution. Current signals were low-pass filtered at 1 kHz and digitized at 2–5 kHz. The signals were corrected for the measured liquid junction potential (−10 mV). EPSC responses to high-frequency stimulation (burst stimulation) were recorded under a blockade of inhibitory synaptic transmissions by applying 20 μm strychnine, an antagonist of glycine receptors, and 100 μm picrotoxin, an antagonist of GABA_A_ receptors. The membrane potential of a recorded neuron was held at −75 mV during the recordings. Burst stimulation with 20 cathodal square-wave pulses (40–50 μA, 100 μs in duration) was applied at 30 s intervals in the vicinity of a recorded neuron using a glass micropipette that was filled with the extracellular solution. The site where the current response of the neuron was the largest was determined as an appropriate stimulation site. In this experiment, the distance between a recorded neuron and the stimulation electrode was 40–120 μm. Three current traces were recorded from each neuron. The current responses of AMPA receptors and NMDA receptors were investigated by local application of 1 mm kainic acid (KA) or 1 mm NMDA to the soma of a recorded neuron, respectively. Different micropipettes were filled with KA or NMDA and were applied separately via pressurized air (30 psi, 5–8 ms) using a pneumatic PicoPump (catalog #PV820, WPI; RRID:SCR_008593). The tips of the micropipettes were maintained at the sites where the largest current responses were recorded. At the beginning of the recording, the reversal potential at which the application of KA induced no current deflection from baseline was determined. Thereafter, the current responses of AMPA receptors at holding potentials of −60 and +40 mV were recorded to determine the rectification index (RI; Ozawa et al., 1991). The current responses of AMPA and NMDA receptors were recorded at holding potentials of −70 and +40 mV, respectively. To monitor the series resistance during the recordings, a short voltage pulse (−10 mV, 100 ms) was applied before agonist application. When the series resistance, which was routinely compensated by 60%, changed by >20% during the recordings, the data were discarded.

### Cobalt uptake

To histologically determine the neurons that express CP-AMPA receptors, we performed a Co^2+^ uptake procedure according to the method of Shimshek et al. (2006). In brief, 400-μm-thick frontal slices were cut as described above. After the recovery of slices in the interface-type chamber, they were incubated in a low-Na^+^ Krebs’ solution with 0.5 μm tetrodotoxin (TTX) and 50 μm D-(-)-2-amino-5-phosphonopentanoic acid (APV) for 15 min. This Krebs’ solution was composed of the following (in mm): 135 sucrose, 50 NaCl, 2.5 KCl, 1.25 NaH_２_PO_４_, 2 MgCl_2_, 0.5 CaCl_2_, 26 NaHCO_3_, and 25 glucose. As a negative control, slices were incubated in Krebs’ solution containing TTX, APV, and 20 μm 2,3-Dioxo-6-nitro-1,2,3,4-tetrahydrobenzo[f]quinoxaline-7-sulfonamide (NBQX) disodium salt. The slices were transferred to Krebs’ solution containing TTX, APV, 20 μm KA, and 1.5 mm CoCl_2_, and incubated for 20 min. After KA-induced CO_2_^+^ loading, the slices were incubated in Krebs’ solution without divalent ions containing 5 mm EDTA for 10 min and further washed with Krebs’ solution without divalent ions. Intracellular Co^2+^ was precipitated by incubating in Krebs’ solution without divalent ions containing 0.12% NH_4_S for 5 min and washing with Krebs’ solution without divalent ions. Thereafter, the slices were fixed with 4% paraformaldehyde in 0.1 m PBS and kept in the fixative for 1–3 d at 4°C. The slices were further cut to a thickness of 50 μm with a sliding microtome (TU-213, Yamato Kohki). For silver intensification, the slices were incubated in Na_2_WO_4_ (2% in water) for 10 min and then in a 20 ml developer solution (16 ml of AgNO_3_ solution containing 1% Triton X-100, 7.5% CH_3_COOH, 30.3 mm Na-acetate, 2.94 mm AgNO3, 2 ml of 5% Na_2_WO_4_, and 2 ml of 0.25% ascorbic acid) in the dark for 7 min. The slices were washed with Na_2_WO_4_, dried, and coverslipped with Entellan (Merck; RRID:SCR_001287). Labeled neurons were observed using a light microscope (model BX60, Olympus).

### Drugs

NMDA, KA, APV, 1-naphthyl acetyl spermine (NAS), and NBQX were purchased from Tocris Bioscience (RRID:SCR_003689); strychnine hydrochloride, flufenamic acid (FFA), and QX-314 were purchased from Sigma-Aldrich (RRID:SCR_008988); NH_4_S was purchased from Strem Chemicals; and other drugs were purchased from Wako Pure Chemical Industries (RRID:SCR_013651). The agonists and antagonists, with the exception of FFA, were dissolved in water (1000 times the final concentration) and stored as stock solutions at −20°C before being diluted in the oxygenated extracellular solution. FFA stock was prepared in DMSO (2000 times the final concentration). KA and NMDA used for local application were stored at a concentration of 20 mm dissolved in water and were diluted to 1 mm with the extracellular solution before use.

### Data analysis

Offline analysis was performed with AxoGraph X software (RRID:SCR_014284) and KaleidaGraph (RRID:SCR_014980). EPSCs were determined when the peak of the inward current was larger than three times the SD of the baseline noise before burst stimulation. The duration of the increased EPSC frequency was defined as the time period from when burst stimulation was terminated to when the averaged value of three adjacent bins (corresponding to 300 ms) became equal to or smaller than the average baseline EPSC frequency before burst stimulation. The EPSC frequency after burst stimulation was measured from recordings of 1 s after burst stimulation. The RI was calculated using the formula: RI = (*I*_+40_/+40)/(*I*_−60_/−60), where *I*_+40_ and *I*_−60_ represent the amplitude of KA-induced currents at membrane potentials of +40 and −60 mV from the reversal potential, respectively. The rectification properties are approximately separated by an RI value of 1; RI < 1 and RI ≥ 1 indicate current responses with inwardly and outwardly rectifying properties, respectively (Ozawa et al., 1991). The charge transfer of NMDA receptor-mediated currents was estimated as the area of the currents for 10 s following the puff application. The charge transfer was normalized by the input capacitance that was estimated based on the current induced by a 10 mV voltage step from a holding potential of −70 mV. All values are shown as the mean ± SD, and the error bars in the figures represent the SD. The number (*n*) refers to the number of neurons analyzed, unless otherwise noted. The statistical analysis was performed using unpaired or paired Student’s *t* tests for normally distributed data. For data that did not follow a normal distribution, the Mann–Whitney test and the Wilcoxon signed-rank test were used for unpaired and paired data, respectively. Differences in the distributions between the groups were tested using the Kolmogorov–Smirnov test. Data normality was determined using the Shapiro–Wilk test. These analyses were performed using StatView software (version 5.0, Hulinks) and JMP software (version 6.0.2; RRID:SCR_014242). A *post hoc* power analysis was performed using G*Power3 software (version 3.1.9.4, http://www.gpower.hhu.de/; RRID:SCR_013726; Faul et al., 2007). Statistical significance was determined at the level of *p* < 0.05. The results of the statistical analyses are shown in Table 1.

**Table 1:** Statistical test

Label	Parameter (unit)	Bivariate	Cell#	Mean	SD	Distribution, ***p*** value (type of test)	Power (α = 0.05)
A	Duration of PHN neurons (s)	Control NAS	8 8	2.6 0.9	0.8 0.3	Normal, *p* = 0.0008 (paired *t* test)	1.000
B	Duration of INC neurons (s)	Control NAS	8 8	2.1 1.8	0.6 0.6	Non-normal, *p* = 0.00107 (Wilcoxon signed-rank test)	1.000
C	EPSC rate of PHN neurons (event/s)	Control NAS	8 8	30.3 16.0	6.7 8.5	Normal, *p* = 0.0005 (paired *t* test)	1.000
D	EPSC rate of INC neurons (event/s)	Control NAS	8 8	32.1 25.7	13.6 11.8	Normal, *p* = 0.0011 (paired *t* test)	0.999
E	Reduction in duration by NAS (%)	PHN INC	8 8	62.2 16.2	15.4 8.9	Non-normal, *p* = 0.0008 (Mann–Whitney test)	1.000
F	Reduction in EPSC rate by NAS (%)	PHN INC	8 8	48.3 21.8	21.4 10.9	Normal, *p* = 0.0005 (unpaired *t* test)	0.906
G	Distribution of RI	PHN INC	48 45	0.88 1.00	0.21 0.21	Normal, *p* = 0.01 (unpaired *t* test)	0.833
H	Duration of PHN neurons (s)	Control APV	10 10	2.1 1.9	0.8 0.8	Non-normal, *p* = 0.1359 (Wilcoxon signed-rank test)	0.524
I	Duration of INC neurons (s)	Control APV	10 10	1.7 0.7	0.4 0.3	Normal, *p* < 0.0001 (paired *t* test)	1.000
J	EPSC rate of PHN neurons (event/s)	Control APV	10 10	22.1 19.8	8.5 8.1	Normal, *p* = 0.0594 (paired *t* test)	0.637
K	EPSC rate of INC neurons (event/s)	Control APV	10 10	21.7 14.6	6.1 5.3	Normal, *p* = 0.0004 (paired *t* test)	1.000
L	Reduction in duration by APV (%)	PHN INC	10 10	10.8 57.6	11.2 13.1	Normal, *p* < 0.0001 (paired *t* test)	1.000
M	Reduction in EPSC rate by APV (%)	PHN INC	10 10	14.7 33.2	13.1 14.2	Normal, *p* = 0.0071 (paired *t* test)	0.899
N	NMDA/AMPA ratio	PHN INC	44 38	0.56 0.82	0.45 0.44	Non-normal, *p* < 0.0001 (Mann–Whitney test)	0.841
O	NMDA charge transfer (pC/pF)	PHN INC	44 38	11.8 19.2	5.6 8.1	Non-normal, *p* < 0.0001 (Mann–Whitney test)	0.999
P	Duration of PHN neurons (s)	Control FFA	8 8	2.4 1.2	0.6 0.5	Normal, *p* = 0.0031 (paired *t* test)	0.990
Q	Duration of INC neurons (s)	Control FFA	8 8	2.7 2.1	0.7 0.7	Non-normal, *p* = 0.0116 (Wilcoxon signed-rank test)	0.963
R	EPSC rate of PHN neurons (event/s)	Control FFA	8 8	29.0 14.3	10.0 4.8	Normal, *p* = 0.0081 (paired *t* test)	0.948
S	EPSC rate of INC neurons (event/s)	Control FFA	8 8	26.3 21.3	6.7 5.6	Normal, *p* = 0.0151 (paired *t* test)	0.900
T	Reduction in duration by FFA (%)	PHN INC	8 8	47.7 22.0	22.3 13.3	Normal, *p* = 0.0143 (unpaired *t* test)	0.844
U	Reduction in EPSC rate by FFA (%)	PHN INC	8 8	46.1 18.4	21.5 13.4	Normal, *p* = 0.008 (unpaired *t* test)	0.902

## Results

**Figure 1.**
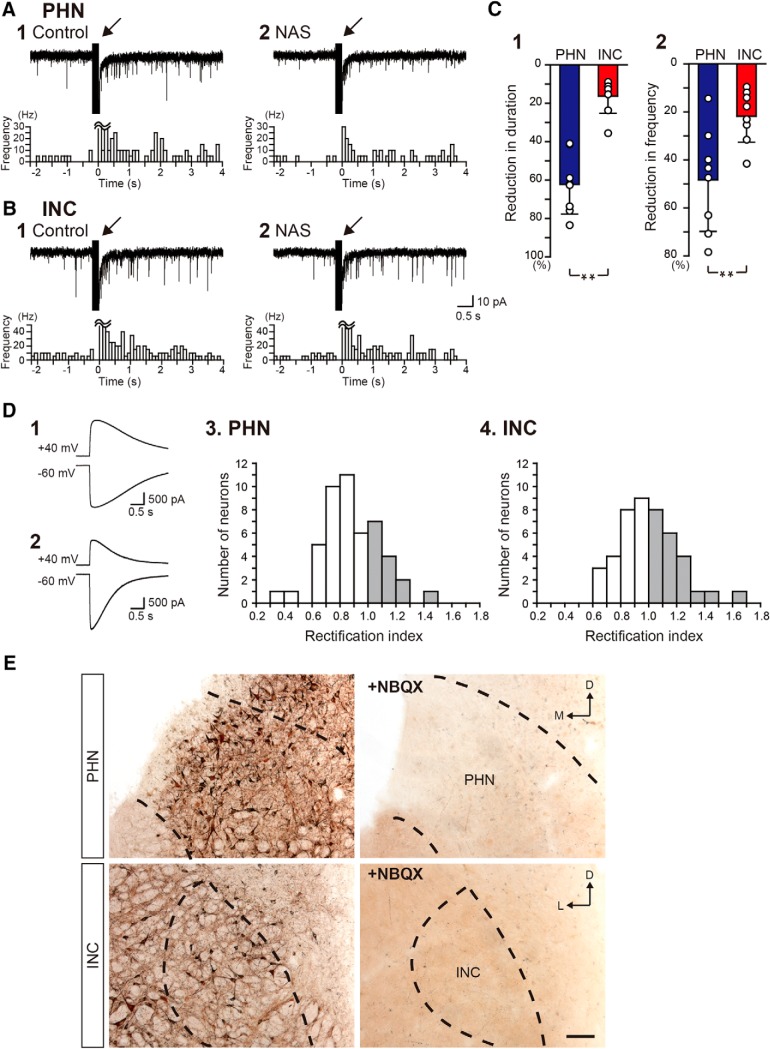
Difference in the expression of CP-AMPA receptors in PHN and INC neurons. ***A***, ***B***, EPSC responses of a PHN neuron (***A***) and an INC neuron (***B***) to burst stimulation before (***A1***, ***B1***) and during (***A2***, ***B2***) the application of 50 μm NAS. The arrow indicates the artifact induced by the burst stimulation. Bottom, Histograms showing the averaged EPSC frequency against time. The width of the histogram bins is 100 ms. ***C1***, ***C2***, Comparisons of the percentage reduction in EPSC duration (***C1***) and frequency (***C2***) caused by NAS between PHN and INC neurons. Asterisks indicate a significant difference between groups (**p < 0.01). ***D***, Current responses of two different INC neurons (***D1***, ***D2***) to puff application of 20 mm kainate at holding potentials of −60 and +40 mV. Histograms of PHN neurons (***D3***; *n* = 48) and INC neurons (***D4***; *n* = 45) exhibiting different RI values. Gray bars indicate an RI > 1. ***E***, Kainate-induced Co^2+^ uptake in the PHN (top) and the INC (bottom). Darkly stained cells indicate the cells that took Co^2+^ into their body via Ca^2+^-permeable AMPA receptors. Right panels show the negative control that was obtained in the presence of NBQX. The dashed lines indicate the approximate regions of the PHN and the INC.

Before investigating the mechanisms of INC excitatory network activation, we revalidated that CP-AMPA receptors had a weaker contribution to the sustained EPSC responses of INC neurons than PHN neurons in Long–Evans rats. Figure 1, *A* and *B*, shows EPSC responses of a PHN neuron and an INC neuron, respectively, to burst stimulation before (Fig. 1*A1*,*B1*) and during (Fig. 1*A2*,*B2*) the application of 50 μm NAS, an open-channel blocker of CP-AMPA receptors (Asami et al., 1989; Takazawa et al., 1996; Koike et al., 1997; Twomey et al., 2018). The burst stimulation (Fig. 1*A*,*B*, arrow) induced an increase in the frequency of spontaneous EPSCs that lasted for several seconds in the PHN and INC neurons. Although NAS application significantly reduced the duration of the sustained EPSC responses and the EPSC frequency for 1 s after burst stimulation in PHN neurons (Table 1, A, C) and INC neurons (Table 1, B, D), marked reductions were observed in the PHN neurons, while only slight reductions were observed in the INC neurons. INC neurons exhibited significantly smaller reductions in EPSC duration and frequency than PHN neurons (Table 1, E, F). These results confirm the finding that CP-AMPA receptors strongly participate in the activation of PHN networks but weakly participate in the activation of INC networks. Neurons that express CP-AMPA receptors show an inwardly rectifying current response property and are estimated according to the RI values (Iino et al., 1990; Hume et al., 1991; Burnashev et al., 1992; Ozawa and Iino, 1993; for review, see Jonas and Burnashev, 1995; Ozawa et al., 1998). We therefore investigated the distribution of neurons that expressed CP-AMPA receptors by estimating the RI from the current responses of PHN and INC neurons to air-puff application of 1 mm KA, which is a nondesensitizing agonist of AMPA receptors (Kiskin et al., 1986; Patneau and Mayer, 1991), at holding potentials of −60 and +40 mV. Figure 1*D1* shows current responses of a neuron, for which the amplitude of current at +40 mV was comparable to that at −60 mV. The RI value of this neuron was >1 (RI = 1.28), indicating that the current responses of this neuron exhibit an outwardly rectifying property. On the other hand, Figure 1*D2* shows the current responses of another neuron, for which the amplitude of current at +40 mV was much smaller than that at −60 mV (RI = 0.67; Fig. 1*D2*), indicating that the current responses of this neuron exhibited an inwardly rectifying property. Histograms of PHN and INC neurons were plotted according to the RI values (Fig. 1*D3*,*4*). The proportion of neurons that exhibited RI < 1 (open bars) was larger in the PHN (70.8%, *n* = 48) than in the INC (53.3%, *n* = 45), and the neuronal distributions based on the RI significantly differed between the PHN and the INC (Table 1, G). This result indicates that more neurons expressing CP-AMPA receptors are present in the PHN than in the INC. To further clarify the presence of neurons that express CP-AMPA receptors both in the PHN and the INC, we performed a KA-induced cobalt uptake experiment (Pruss et al., 1991; Engelman et al., 1999: Shimshek et al., 2006), in which neurons uptake and accumulate Co^2+^ during activation by KA. Using the Co^2+^ uptake procedure, darkly stained neurons were found to be scattered in the PHN and the INC (Fig. 1*E*, left), although neurons were not labeled in the presence of 20 μm NBQX, an AMPA receptor antagonist (Fig. 1*E*, right). This result confirms the presence of neurons that express CP-AMPA receptors not only in the PHN, but also in the INC.

**Figure 2.**
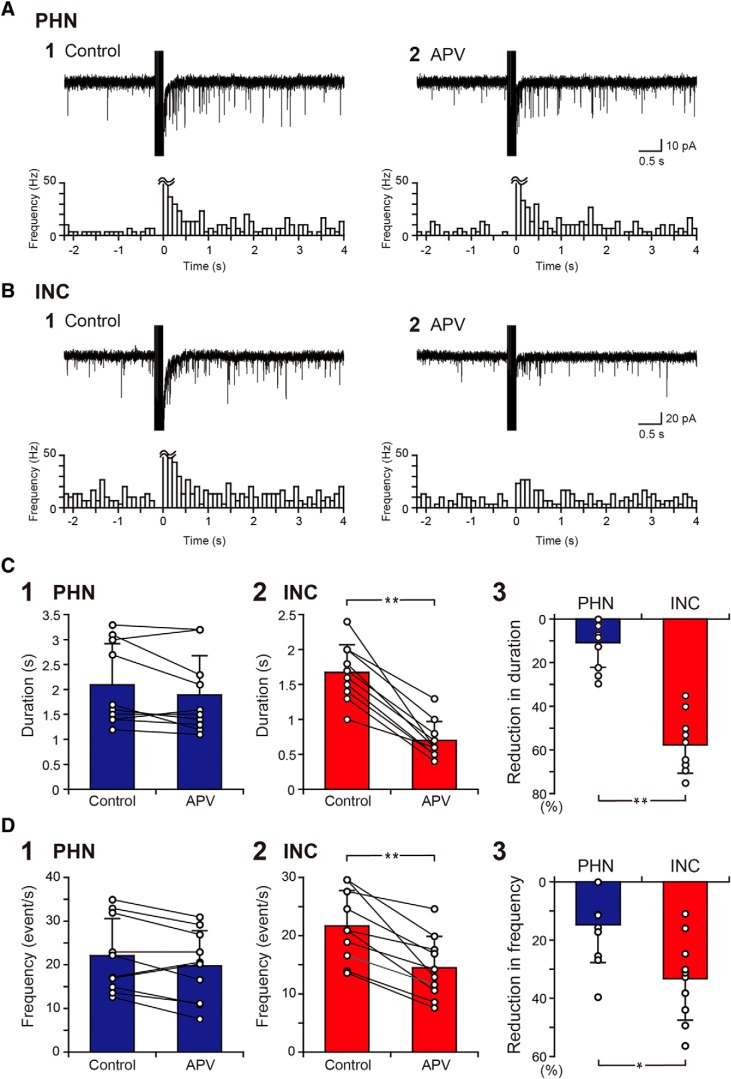
Comparison of the effects of APV on EPSC responses between PHN and INC neurons. ***A1***, ***A2***, EPSC responses of a PHN neuron to burst stimulation before (***A1***) and during (***A2***) the application of 50 μm APV. ***B1***, ***B2***, EPSC responses of an INC neuron to burst stimulation before (***B1***) and during (***B2***) APV application. Bottom, Histograms showing the averaged EPSC frequency against time. The width of the histogram bins is 100 ms. ***C1***, ***C2***, Comparison of the duration of the increased EPSC frequency of PHN (***C1***) and INC (***C2***) neurons before and during APV application. ***C3***, Comparison of the percentage reduction in the duration caused by APV between PHN and INC neurons. ***D1***, ***D2***, Comparison of the frequency of EPSCs after burst stimulation of PHN (***D1***) and INC (***D2***) neurons before and during APV application. ***D3***, Comparison of the percentage reduction in the EPSC frequency caused by APV between PHN and INC neurons. Plots indicate data obtained from individual neurons, and the bar represents the average value. Asterisks indicate a significant difference between groups (*p < 0.05; **p < 0.01).

Our previous study demonstrated that the sustained EPSC responses in the PHN were not significantly affected by APV, an NMDA receptor antagonist (Saito and Yanagawa, 2010). This finding was confirmed by the present study (Fig. 2*A*), in which the EPSC duration and frequency of PHN neurons were not significantly different before and after the application of APV (Fig. 2*C1*,*D1*, Table 1, H, J). However, when the effect of APV on the sustained EPSC responses was tested in INC neurons (Fig. 2*B*), the EPSC duration and frequency were found to be significantly reduced in the presence of APV (Fig. 2*C2*,*D2*, Table 1, I, K). Significantly larger reductions in the EPSC duration and frequency were found in INC neurons than in PHN neurons (Table 1, L, M). These results suggest that NMDA receptors have a strong contribution to the activation of INC networks than to the activation of PHN networks.

**Figure 3.**
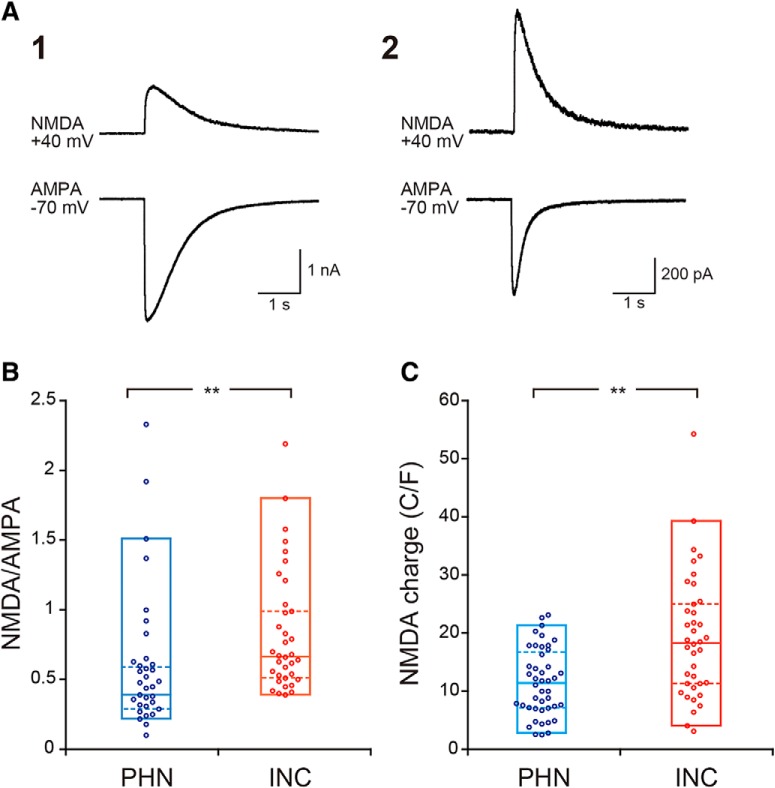
Comparison of NMDA receptor-mediated currents between PHN and INC neurons. ***A***, Current traces of two different INC neurons to puff application of 1 mm kainite at a holding potential of −70 mV and of 1 mm NMDA at a holding potential of +40 mV. The NMDA/AMPA ratios of neurons (***A1***, ***A2***) were 0.39 and 1.26, respectively. ***B***, ***C***, Comparisons of the NMDA/AMPA ratio (***B***) and the charge transfer of the NMDA receptor-mediated currents (***C***) between PHN and INC neurons. Plots indicate data obtained from individual neurons. Boxes indicate percentile plots. The bottom and top of each box represent the 5% and 95% percentiles of the data, respectively. The middle line in the box represents the median value of the data, whereas the lower and upper dashed lines represent the 25th and 75th percentiles of the data, respectively. Asterisks indicate a significant difference between groups (*p* < 0.01).

Based on the stronger effect of APV on INC neurons, we next examined whether current responses mediated via NMDA receptors differed between PHN and INC neurons. To compare NMDA receptor-mediated currents, we analyzed the ratio of NMDA receptor-mediated currents to AMPA receptor-mediated currents (NMDA/AMPA ratio; Fig. 3*A*,*B*). The NMDA/AMPA ratio of INC neurons was significantly higher than that of PHN neurons (Fig. 3*B*, Table 1, N). In addition, the charge transfer mediated via NMDA receptors of INC neurons was significantly larger than that of PHN neurons (Fig. 3*C*, Table 1, O).

**Figure 4.**
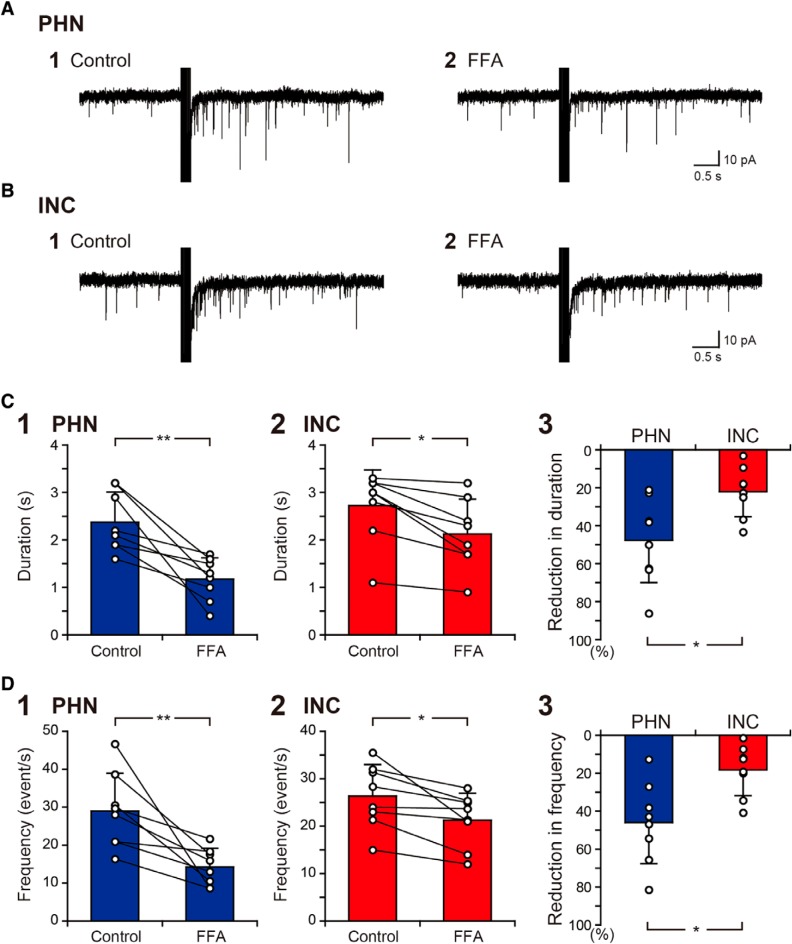
Comparison of the effects of FFA on EPSC responses between PHN and INC neurons. ***A1***, ***A2***, EPSC responses of a PHN neuron to burst stimulation before (***A1***) and during (***A2***) the application of 200 μM flufenamic acid (FFA). ***B1***, ***B2***, EPSC responses of an INC neuron to burst stimulation before (***B1***) and during (***B2***) FFA application. ***C1***, ***C2***, Comparison of the duration of the increased EPSC frequency of PHN (***C1***) and INC (***C2***) neurons before and during FFA application. ***C3***, Comparison of the percentage reduction in the duration caused by FFA between PHN and INC neurons. ***D1***, ***D2***, Comparison of the EPSC frequency after the burst stimulation of PHN (***D1***) and INC (***D2***) neurons before and during FFA application. ***D3***, Comparison of the percentage reduction in the EPSC frequency caused by FFA between PHN and INC neurons. Plots indicate data obtained from individual neurons, and the bar represents the average value. Asterisks indicate a significant difference between groups (*p < 0.05; **p < 0.01).

Our previous study showed that the application of FFA, a blocker of CAN channels, also reduced the sustained EPSC responses of PHN neurons (Saito and Yanagawa, 2010). This finding was confirmed by the present study (Fig. 4*A*), in which the EPSC duration and frequency of PHN neurons were significantly reduced after the application of FFA (Fig. 4*C1*,*D1*, Table 1, P, R). Significant reductions in the EPSC duration and frequency by the application of FFA were also observed in INC neurons (Fig. 4*C1*,*D1*, Table 1, Q, S). However, the reductions in the EPSC duration and frequency were significantly smaller in INC neurons than in PHN neurons (Fig. 4*C3*,*D3*, Table 1, T, U). These results suggest that CAN channels have a weaker contribution to the activation of INC networks than to the activation of PHN networks.

## Discussion

In this study, we investigated the synaptic mechanisms of the sustained activation of excitatory networks that produce position signals in horizontal and vertical oculomotor neural integrators. The activation of excitatory networks in the horizontal integrator was mediated predominantly via CP-AMPA receptors and CAN channels while their activation in the vertical integrator was mediated predominantly via NMDA receptors. Our findings strongly suggest that excitatory networks are activated via different synaptic mechanisms between the horizontal and vertical integrators.

It has been reported that both the PHN and the INC show reciprocal connections with the vestibular nuclei (VNs; Fukushima, 1987; McCrea and Horn, 2006; Leigh and Zee, 2015). The VNs carry eye position signals (Tomlinson and Robinson, 1984; McFarland and Fuchs, 1992), and chemical lesion of the VNs or transection of the connections between the VNs and the integrators induces impairments in gaze holding (Evinger et al., 1977; Cannon and Robinson, 1987; Godaux et al., 1993; Arnold et al., 1999). Therefore, the sustained activity responsible for horizontal and vertical eye position signals may be maintained by reciprocal loops in the PHN–VN and INC–VN networks, respectively (Evinger et al., 1977; Cannon and Robinson, 1987; McFarland and Fuchs, 1992; Sylvestre et al., 2003). Our present and previous studies demonstrated that sustained EPSC responses, indicating local excitatory networks, occurred in reduced preparations in which the connections to the VNs were discontinued (Saito and Yanagawa, 2010; Saito et al., 2017). These findings suggest that recurrent excitatory networks that produce sustained EPSC responses are deployed within the PHN and the INC.

Excitatory networks are inherently leaky. Therefore, when excitatory networks are driven only by fast synaptic transmissions mediated by AMPA receptors, the activity generated by the network is neither sustained nor robust (Seung et al., 2000; Wang, 2001). In the PHN, synaptic transmissions are primarily mediated via AMPA receptors, but Ca^2+^ influx through CP-AMPA receptors activates CAN channels that generate long-lasting plateau depolarizations (Morisset and Nagy, 1999; Di Prisco et al., 2000; Fransén et al., 2006). The prolonged activity generated by CAN channels can persistently and robustly activate excitatory networks. The present physiologic (RI) and histologic (Co^2+^ uptake) analyses demonstrated that neurons that expressed CP-AMPA receptors existed not only in the PHN but also in the INC. The difference in the neuronal distribution between the PHN and the INC according to the RI suggests that the INC has a smaller proportion of neurons that express CP-AMPA receptors than the PHN. Therefore, the weaker contribution of CP-AMPA receptors in INC networks may be attributed to the small proportion of neurons that express CP-AMPA receptors in the INC. Alternatively, it could be suggested that the majority of neurons that express CP-AMPA receptors in the INC do not participate in the excitatory networks responsible for sustained activity. In this study, a difference in the distribution of neurons that expressed CP-AMPA receptors was not clearly detected in Co^2+^ uptake experiments. Because KA stimulation activated the excitatory networks too strongly, all PHN and INC neurons that expressed CP-AMPA receptors may have had maximum uptake of Co^2+^ into their cell bodies. This possible scenario may explain the negative result.

In contrast to the PHN, the sustained EPSC responses in the INC were mediated preferentially via NMDA receptors rather than via a combination of CP-AMPA receptors and CAN channels. NMDA receptors show a slow time course that requires 10–20 ms to reach the peak and decays over hundreds of milliseconds (Edmonds et al., 1995). This slow time course of the receptors can persistently and robustly activate the INC excitatory networks. Comparison of the NMDA/AMPA ratio revealed that the ratio of INC neurons was higher than the ratio of PHN neurons. In addition, the charge transfer mediated via NMDA receptors was larger in INC neurons than in PHN neurons. These results suggest that the expression of NMDA receptors and/or the conductance of expressed NMDA receptors were higher in INC neurons than in PHN neurons. Studies on cortical pyramidal neurons using caged glutamate or voltage-sensitive dye indicated that NMDA-dependent dendritic plateau depolarization participates in the persistent activity of the neurons (Schiller et al., 2000; Milojkovic et al., 2004). In cortical network models, the larger NMDA/AMPA ratio was found to be involved in the stabilization of persistent activity (Wang, 1999; Compte et al., 2000; Tegnér et al., 2002). In the present study, INC neuron dendrites may have been damaged or truncated during tissue slicing. Therefore, whether dendritic plateau depolarization mediated via NMDA receptors indeed occurs in INC neurons remains an open question.

The EPSC duration and frequency were significantly reduced by FFA in INC neurons, although the effects of FFA were significantly smaller in INC neurons than in PHN neurons. This result suggests that CAN channels are partially involved in the activation of INC excitatory networks. Because previous studies have shown the activation of CAN channels by Ca^2+^ entry through NMDA receptors (Di Prisco et al., 2000; Hall and Delaney, 2002; Zhu et al., 2004), the relationship between CAN channels and NMDA receptors is also considered in INC neurons. However, the effects of FFA were smaller than the effects of APV and were comparable to the effects of NAS (Table 1), suggesting that the activation of CAN channels is implicated in CP-AMPA receptors rather than NMDA receptors in INC neurons.

**Figure 5.**
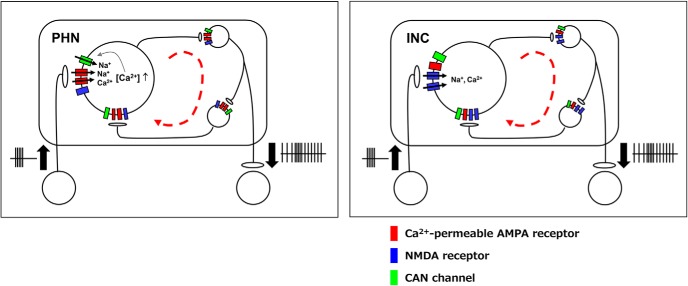
Schematic drawings of the structure of local excitatory circuits in the PHN and the INC. Burst inputs from the premotor areas, such as the pontine reticular formation and the vestibular nucleus, are transformed into tonic outputs to the extraocular motor nuclei by activation of the local excitatory network. Red dashed arrow indicates propagation of excitation.

In the present study, we clarified the different activation mechanisms of local excitatory networks in horizontal and vertical neural integrators. Figure 5 shows the local networks for producing the sustained activity induced in the PHN and the INC. In Figure 5, CP-AMPA receptors and CAN channels are incorporated into all PHN neurons participating in the local networks, and NMDA receptors are incorporated into all INC neurons participating in the networks. However, our previous study on the PHN (Saito and Yanagawa, 2010) showed that the excitatory networks responsible for the sustained EPSC responses included the neurons that did not express CP-AMPA receptors as well as the neurons that expressed the receptors. This finding raises a possibility that some PHN neurons that express CP-AMPA receptors and some INC neurons that express NMDA receptors may play the role of hub neurons that mainly drive sustained activity, although the existence of hub neurons has not been clarified in the PHN or INC. The local excitatory networks themselves may not be sufficient to maintain the activity for gaze holding because the durations of the EPSC responses are shorter than the time constant of the rat neural integrator, which was approximately estimated as a time constant of a centripetal drift after an eye movement to an eccentric direction (Chelazzi et al., 1989). In monkeys, lesions of the commissural inhibitory networks of the neural integrators induced a dramatic reduction in the time constant of the integrators (Arnold and Robinson, 1997), although negative evidence on positive feedback excitation was obtained in goldfish studies (Aksay et al., 2007; Debowy and Baker, 2011). Cerebellar flocculus and paraflocculus inputs modulate the sustained activity of the neural integrators in a positive feedback manner (Zee et al., 1981; Leigh and Zee, 2015). In addition, the sustained depolarization is induced by cholinergic transmissions mediated via muscarinic (Navarro-López et al., 2004, 2005) and nicotinic receptors (Zhang et al., 2016, 2018) in PHN neurons. These modulations of neural activity, in addition to local excitatory networks, can produce the tonic activity sufficient for keeping eyes to an eccentric direction for a moment.

## Synthesis

Reviewing Editor: Christian Hansel, University of Chicago Decisions are customarily a result of the Reviewing Editor and the peer reviewers coming together and discussing their recommendations until a consensus is reached. When revisions are invited, a fact-based synthesis statement explaining their decision and outlining what is needed to prepare a revision will be listed below. The following reviewer(s) agreed to reveal their identity: Emre Aksay. Note: If this manuscript was transferred from JNeurosci and a decision was made to accept the manuscript without peer review, a brief statement to this effect will instead be what is listed below.

The findings are of importance for efforts to understand the mechanisms of temporal integration and persistent firing. The manuscript presents an advancement in the current understanding of the oculomotor integrator circuit. In it, the authors detail differences in the recurrent excitation mechanism between the horizontal and vertical integrators. The results and the methods are well presented and thorough. The discussion, however, is very misleading and argues for differences in the putative mechanism that were not presented in the results. The idea of hub neurons made in the discussion can be supported by reconstruction the morphology of the neurons that were recorded. The authors claim that the time constants are short due to the lack of inputs from the contralateral side (because of the slice prep.) and cite key paper to support this claim. However, they omit citing another paper (Debowy et. al, 2011) that shows that midline lesions do not affect the time constants.

Major comments:

The paper by Saito and Sugimura presents evidence through slice physiology in the rodent model that a contributing synaptic mechanism for temporal integration at the Interstitial Nucleus of Cajal (INC), a key part of the neural integrator for vertical eye movements, is feedback through NMDA receptors. They also confirm their earlier findings that NMDA mediated inputs do not contribute significantly in the Nucleus Prepositus Hypoglossi (NPH), a key part of the neural integrator for horizontal eye movements, where instead Calcium-Activated Nonselective (CAN) cation currents play a more meaningful role. The experiments are well done and the work is presented in a straightforward manner. The findings are of importance for efforts to understand the mechanisms of temporal integration and persistent firing. I suggest a more careful revision of the discussion that makes claims that are supported by the evidence presented here.

  Minor comments:  - Please describe the expected damage from slice preparation to the dendritic arbor of cells in the NPH and INC.  - In Figure 1, please plot the event rate vs time as well as the frequency and duration values as in Figure 2  - The cobalt staining results need to be quantified and properly normalized by the total neuron number in the visualized region. Total counts could be acquired through a nuclear stain.

## Author Response

Manuscript Instructions

- The sex of the species studied is not mentioned in the materials and methods section. Please mention ‘male’, ‘female’, ‘x males and x females’, ‘of either sex’.

Reply: We have added the statement of ‘of either sex’ to the text (Page 6, 5th line from the bottom)

---------------------------------------------

Synthesis of Reviews:

Computational Neuroscience Model Code Accessibility Comments for Author (Required):

N/A

Significance Statement Comments for Author (Required):

N/A

Comments on the Visual Abstract for Author (Required):

N/A

Synthesis Statement for Author (Required):

The findings are of importance for efforts to understand the mechanisms of temporal integration and persistent firing. The manuscript presents an advancement in the current understanding of the oculomotor integrator circuit. In it, the authors detail differences in the recurrent excitation mechanism between the horizontal and vertical integrators. The results and the methods are well presented and thorough. The discussion, however, is very misleading and argues for differences in the putative mechanism that were not presented in the results. The idea of hub neurons made in the discussion can be supported by reconstruction the morphology of the neurons that were recorded. The authors claim that the time constants are short due to the lack of inputs from the contralateral side (because of the slice prep.) and cite key paper to support this claim. However, they omit citing another paper (Debowy et. al, 2011) that shows that midline lesions do not affect the time constants.

Major comments:

The paper by Saito and Sugimura presents evidence through slice physiology in the rodent model that a contributing synaptic mechanism for temporal integration at the Interstitial Nucleus of Cajal (INC), a key part of the neural integrator for vertical eye movements, is feedback through NMDA receptors. They also confirm their earlier findings that NMDA mediated inputs do not contribute significantly in the Nucleus Prepositus Hypoglossi (NPH), a key part of the neural integrator for horizontal eye movements, where instead Calcium-Activated Nonselective (CAN) cation currents play a more meaningful role. The experiments are well done and the work is presented in a straightforward manner. The findings are of importance for efforts to understand the mechanisms of temporal integration and persistent firing. I suggest a more careful revision of the discussion that makes claims that are supported by the evidence presented here.

Reply:

Thanks to the reviewer's suggestion, we have noticed that the statements in the previous version may provide the impression that the existence of hub neurons has already been clarified in the integrator networks. As the reviewer suggested, evidence of morphological and physiological connections to surrounding neurons is needed to claim the existence of hub neurons, and this evidence has not been obtained in the PHN or INC. Our previous study on the PHN (Saito and Yanagawa 2010) showed that the excitatory networks responsible for the sustained EPSC responses included neurons that did not express CP-AMPA receptors as well as neurons that expressed the receptors. This finding raises a possibility that some PHN neurons that express CP-AMPA receptors and some INC neurons that express NMDA receptors may play a role of hub neurons. We have modified the statements regarding hub neurons in order to not mislead readers (Page 22, line 1 -10).

A study using monkeys demonstrated that lesions of the commissural inhibitory networks of the neural integrators induced a dramatic reduction in the time constant of the integrators (Arnold and Robinson 1997). This finding suggests that positive feedback excitation through a mutual inhibition between the bilateral integrator regions is one of the mechanisms that maintain the activity of integrator networks. However, as the reviewer pointed out, negative evidence on positive feedback excitation has been obtained in goldfish studies. We have added this finding to the Discussion (Page 22, the last two lines).

Minor comments:

Please describe the expected damage from slice preparation to the dendritic arbor of cells in the NPH and INC.

Reply:

In the previous version, we stated that NMDA-dependent dendritic plateau depolarization of cortical pyramidal neurons may occur in INC neurons. However, the reviewer has expressed a concern about damage to the dendrites in our slice preparations. We agree with the reviewer that some dendrites of INC neurons may have been damaged or truncated during slicing. Therefore, whether dendritic plateau depolarization mediated via NMDA receptors indeed occurs in INC neurons remains an open question. We have added this statement to the text (Page 21, line 1 - 4).

In Figure 1, please plot the event rate vs time as well as the frequency and duration values as in Figure 2.

Reply:

In accordance with the reviewer's suggestion, we have added histograms showing the relationship between the event rate and the time to Figure 1.

The cobalt staining results need to be quantified and properly normalized by the total neuron number in the visualized region. Total counts could be acquired through a nuclear stain.

Reply:

To confirm the existence of neurons that express CP-AMPA receptors by another analysis in addition to the electrophysiological RI analysis, we performed a Co2+- uptake analysis. Our finding confirmed the existence of the neurons in both the PHN and INC.

In accordance with the reviewer's suggestion, we estimated the percentage of neurons that expressed CP-AMPA receptors. Because nuclear staining without an immunohistochemical procedure (for example, using DAPI) labels not only nuclei of neurons but also nuclei of other cells including glia, we performed Nissl staining using NeuroTrace (Invitrogen) to stain neurons. However, the Nissl staining was incomplete in sections in which the Co2+- uptake procedure was performed. Therefore, we performed Nissl staining using a section not exposed to the Co2+- uptake procedure of approximately the same location as the section exposed to the procedure. The neuron counts were performed in Co2+- uptake sections and Nissl-stained sections separately. In the PHN, there were 100.3 ± 58.5 (n = 3 sections) and 220.0 ± 38.2 (n = 2 sections) Co2+-labeled neurons and Nissl-stained neurons, respectively. In the INC, there were 48.7 ± 35.8 (n = 3 sections) and 118.0 ± 14.1 (n = 2 sections) Co2+-labeled neurons and Nissl-stained neurons, respectively. From these data, the percentage of Co2+-labeled neurons in the PHN and the INC were 45.6% and 41.2%, respectively. However, these values were obtained by an extremely rough analysis; therefore, we have not added them to the text to prevent these values from being taken out of context. We would like to develop a new method to precisely estimate the percentage of Co2+-labeled neurons in the PHN and INC in the future.
